# Advances in Parasite Control in Africa: From Basic Science to Translation

**DOI:** 10.1155/2012/158039

**Published:** 2012-07-19

**Authors:** Francisca Mutapi

**Affiliations:** Institute of Immunology and Infection Research, School of Biological Sciences, University of Edinburgh, Edinburgh EH9 3JT, UK

Parasitology as a subject has a long history in Africa both in the medical and veterinary fields ranging from the identification of parasites to the development of interventions. For example, both of the parasites of major public health concern in tropical countries were discovered in Africa despite occurring in other parts of the world. Thus, the *Plasmodium *parasite causing malaria was discovered by Charles Louis Alphonse Laveran, a French army surgeon stationed in Constantine, Algeria, on the 6th of November 1880, a discovery for which he was awarded the Nobel Prize in 1907. The livestock protozoan *Theileria parva*, which causes East Coast Fever in cattle, was also discovered in Africa by Arnold Theiler and Charles Lounsbury in South Africa in 1904. In 1851, Theodor Bilharz, discovered the blood fluke parasite (*Schistosoma haematobium) *which causes bilharzia or snail fever, during a postmortem examination at the Kasr-el-Aini hospital in Cairo.

Since these discoveries, there have been concerted efforts worldwide to find interventions ranging from drugs used to kill parasites in human hosts (quinine; artemisinins for *Plasmodium *and the antihelminthics metrifonate; oxamniquine and praziquantel for schistosomes), drugs to kill vectors/intermediate hosts (dichlorodiphenyltrichloroethane (DDT) for *Plasmodium *mosquito vectors; acaricides for tick vectors of *Theileria *and copper sulphate/niclosamide for killing schistosome snail hosts), and barriers to prevent the contact of human hosts and infective stages (e.g., bed nets for *Plasmodium*).

With the description of life cycles of not only these parasites but other protozoans (e.g., Giardia) and helminths (e.g., hookworms), the importance of sanitation, hygiene and clean water became apparent, and these lead to inventions such as the Blair toilet, a simple pit latrine developed in Zimbabwe and safe drinking water wells. These developments hold true to the observation by the Roman Scholar and Scientist Pliny the Elder (*23 AD–79 AD*) that “there is always something new out of Africa,” albeit it is in a different context.

With technological advances in this postgenomic era, collaborations between scientists in different institutions and countries in Africa as well as collaborations between African scientists and those in other continents (loosely termed north-south collaborations) have allowed scientists to conduct cutting-edge target-species-oriented research. The development and fostering of these collaborations and partnerships themselves have been as vital as the technological advances in efforts to control parasitic diseases. Thus, for example, work on RTS,S, the world's most clinically advanced malaria vaccine candidate [1], has included 11 clinical trial sites in seven African countries: Burkina Faso, Gabon, Ghana, Kenya, Malawi, Mozambique, and Tanzania with over 15,000 participants, and industrial (GlaxoSmithKline), research (PATH Malaria Vaccine Initiative), and NGO (Bill & Melinda Gates Foundation) partners involved in over 20 years of work. Independent of the outcome of the clinical trials, this level of collaboration sets a precedent for all future programmes for better interventions for parasitic diseases in Africa. The basic scientific work for such collaborative studies is already currently being conducted in Africa as exemplified by work published in this special issue. These include detection of parasites in wildlife as reported by Munang'andu et al. Parasite surveillance of wildlife is important not only for monitoring parasites that affect domestic animals but also for identifying emerging zoonotic parasites and pathogens. Once parasites are identified on hosts, there is need for reliable diagnostic methods which are applicable in clinical and field settings as appropriate. Clive Shiff sets out the case for the need for definitive diagnosis for urogenital schistosomiasis and the pathology arising during chronic infection. The diagnostic method focused on by Shiff relies on recent advances in molecular biology, polymerase chains reaction (PCR), which detects parasite fragments in urine. Wumba et al. also use PCR to detect the presence of *Enterocytozoon bieneusi*, a microsporidia parasite that has become an important opportunistic infection affecting AIDS patients [2]. Opportunistic parasites/pathogens represent one of the clearest demonstrations of coinfections that occur in human and animal populations. The effect of one infection on the immune system can influence the susceptibility of a host to infection by a second pathogen (as occurs in the case of HIV immune-compromised individuals), disease progression (HIV progression during helminth infection [3]), and pathological processes (liver pathology in children infected with malaria parasites and schistosomes [4]). Perhaps counterintuitively, some infections can protect against infection by others, for example, the helminth *Ascaris lumbricoides *can protect against *Plasmodium falciparum *infection [5] or against pathology from another parasite (e.g., intestinal helminthes can protect against anemia during an acute malarial attack by *P. vivax *[6]). These examples show that these associations are both complex and context-dependent. One of the reasons for such complexity is heterogeneity in various host attributes such as genetic background and nutritional status. The study by Reilly et al. focuses on micronutrients which play an important role in the development and function of the immune system. This study highlights the association between micronutrients and cytokines (mediators of immune responses) in schistosome-infected people.

As mentioned earlier, environmental factors such as water sources and toilets affect transmission of several parasites. This means that successful control strategies must address the contribution of these factors. In his article on schistosome control strategies, Moses Chimbari highlights the importance of an integrated and multisectorial approach for successful parasite control using examples from Zimbabwe. Gosh et al. extend this concept by highlighting the importance of socioeconomic, political, and cultural aspects in successful control of parasitic diseases.

All the studies published in this special issue illustrate the importance of multidisciplinary approaches in basic research to develop effective interventions as well as an understanding of the interactions between environmental, socioeconomic, political, and cultural influences on transmission and delivery of the interventions for successful and sustainable control. For several parasites, these issues are already known and well understood, and there are already efficacious control methods available, the *immediate challenge *is to make effective use of this knowledge and the tools currently available. The *future challenge *is to keep ahead of the parasites by effective surveillance for (1) emerging parasites and (2) changes in the parasite phenotypes and their frequencies in response to control efforts and then developing control measures that can adapt to these parasite changes.

*Francisca Mutapi* *Francisca Mutapi*
